# The effects of different biochars on *Caenorhabditis elegans* and the underlying transcriptomic mechanisms

**DOI:** 10.1371/journal.pone.0284348

**Published:** 2023-09-22

**Authors:** Yixuan Chen, Xinrui Wang, Jie Li, Zhiwen Wang, Tingting Song, Xin Lai, Guilong Zhang, Weibin Ruan

**Affiliations:** 1 Agro-Environmental Protection Institute, Ministry of Agriculture and Rural Affairs, Tianjin, China; 2 College of Agronomy, Shenyang Agricultural University, Shenyang, China; 3 College of Resources and Environment, Northeast Agricultural University, Harbin, China; 4 Institute of Environment and Sustainable Development in Agriculture, CAAS, Beijing, China; 5 College of Life Sciences, Nankai University, Tianjin, China; East Carolina University, UNITED STATES

## Abstract

Different biochars have diverse properties, with ambiguous effects on soil nematodes. This study investigated how aspen sawdust (ABC), bamboo powder (BBC), maize straw (MBC) and peanut-shell biochars (PBC) affected *Caenorhabditis elegans* via culture assays and RNA-seq analysis. The results showed that biochars derived from different agricultural materials varied significantly in physicochemical properties, and PBC produced more volatile organic compounds (VOCs) to attract C. *elegans* than ABC, BBC and MBC. Moreover, worms in ABC experienced the worst outcomes, while worms in PBC experienced milder impacts. Nematode body length decreased to 724.6 μm, 784.0 μm and 799.7 μm on average in ABC, BBC and MBC, respectively, compared to the control (1052 μm) and PBC treatments (960 μm). The brood size in ABC, MBC, BBC and PBC decreased 41.1%, 39.4%, 39.2% and 19.1% compared to the control, respectively. Furthermore, the molecular mechanisms of biochar-induced developmental effects on C. *elegans* were explored. Although several differentially expressed genes (DEGs) were different among the four biochars, worm phenotypic changes were mainly related to *col* genes (*col-129; col-140; col-40; col-184*), *bli-6*, *sqt-3*, *perm-2/4*, *cdk-8*, *daf-16* and *sod-1/2/5*, which are associated with cuticle collagen synthesis, eggshell formation in postembryonic growth and rhythmic processes. Our study suggests that different properties of biochars could be crucial to soil nematodes, as well as the worms’ biochemical changes are important for the health in agriculture soil.

## 1. Introduction

With the rapid increase in the production of agricultural wastes in China, the recycling and utilization of these potential biomass resources have become crucial for alleviating environmental pollution and improving soil fertility [1]. In recent decades, biochar has been widely researched for its advantages in recycling agricultural wastes [2]. Studies claim that the application of biochars made from various agricultural wastes will not only enhance the recycling rate of wastes but also improve the properties and structure of soil [3]. Nevertheless, it is necessary to explore how biochar changes the soil biota since the application of biochar changes soil properties [4, 5].

Nematodes play a crucial role in soil biota systems, being dominant in abundance, having a diversity of life cycles, and occurring at various trophic levels [6]. Previous studies have reported the interactions between biochar and soil nematodes. The main results can be concluded as follows: i) biochar improves nutrient supply and habitat conditions, which would support more microbivorous nematodes and exert competitive pressure on other types of nematodes as well as change the community composition of soil nematodes [7, 8]. ii) Biochars improve soil porosity and root growth to inhibit the growth of some harmful phytophagous nematodes [9]. iii) In recent years, researchers have also stated that some substances in biochar can disturb the movement and growth of some nematodes [10, 11]. In general, those studies mainly focused on assessing the effect of biochar addition on soil nematode community abundance, structure and biodiversity or focused on certain plant parasitic nematodes. Few studies have tested the direct impacts of biochar on nematodes. Additionally, researchers further concluded that the properties of biochars vary widely due to the marked differences in the raw materials used [12, 13], so clarifying and comparing the soil nematode response to different biochars is essential to reveal the underlying mechanisms of biochar interactions with soil fauna.

Contemporary studies also demonstrated that the composition of different raw materials resulted in various yields and characteristics of biochars. A higher lignin content can result in a higher biochar yield; the percentage of lignocellulosic biomass (cellulose, hemicellulos, lignin) or minerals in biochar can result in different pH values, pore compositions or ash contents [14]. According to Lebrun [15], even biochars made from different layers of oak tree trunk (bark, sapwood, heartwood) have different properties. These differences between biochars caused different effects on the abundance, activity and feedback of soil nematodes. Total nematode abundance and faunal activity were different under wheat or corn biochar application [7, 16]. Biochars derived from rice husk and sawdust showed negligible toxic effects on *C*. *elegans*, while *Acorus calamus* (a wetland plant) biochar showed significant toxicity toward the worms [17]. Phenolic and polycyclic aromatic hydrocarbons (PAHs) [18] or persistent free radicals [11] from some biochars resulted in worm growth inhibition or avoidance, which implied that some biochars may have significant negative effects on the growth of nematodes. Based on these phenomena, the effects of different biochars on soil nematodes vary. However, few studies have investigated the response mechanisms of nematodes exposed to biochars produced by different raw materials.

Maize, aspen, bamboo and peanut are very important wood and agricultural resources in China [19, 20]. Moreover, these wastes or byproducts are difficult to degrade, recycle and manage. The direct use of these wastes may not only cause many problems in field production, such as unfavorable root fixation, more disease for the next crop, and lower seedling survival [21], but also result in high costs during recycling [22]. Therefore, the application of biochar derived from these wastes to arable land is a novel and convenient approach. One of the most highly debated points prior to biochar application might be how different biochars affect the development of soil nematodes.

*C*. *elegans* is a model organism in the fields of molecular biology, developmental biology and environmental toxicology because of its well-studied genetic background. Therefore, it is a perfect tool to investigate the effect of biochars on nematodes at the individual and molecular levels. Therefore, the aim of our study was 1) to compare the differences among the four biochars (peanut shell biochar, PBC; aspen sawdust biochar, ABC; bamboo powder biochar, BBC; maize straw biochar, MBC), including their physico-chemical properties, VOCs and PAHs. 2) Assessment of the body length, SOD activity, reproduction and lifespan of *C*. *elegans* exposed to different types of biochar was also performed. 3) Furthermore, RNA-seq was performed to obtain transcript sequences of the worms. Additionally, differentially expressed genes (DEGs) related to differential phenotypes revealed genome-wide transcript changes in *C*. *elegans* in response to biochar exposure.

## 2. Materials and methods

### 2.1. Biochar preparation and characterization

Four different biochars were produced by grinding maize straw (M), aspen sawdust (A), bamboo powder (B) and peanut shells (P) through a 0.85 mm sieve. These raw materials were pyrolyzed at 500°C without oxygen (N_2_) for 120 min in a muffle furnace (HBYQ 2200) and then cooled to room temperature and passed through a 1.5 mm sieve. The lignocellulosic biomass of the materials was tested with an ANKOM A2000i fiber analyzer through Soest [23] detergent analysis and calculated by the difference between neutral detergent fiber (NDF), acid detergent fiber (ADF), lignin and crude fiber.

The porosity and specific surface area, pH value and relative atomic ratio of these biochars were determined by SEM TM-1000 (Hitachi, Japan), an MP511 lab pH meter (San-xin, China) and XPS Escalab 250Xi (Thermo Scientific, USA). The ash content was measured by the China National Academy of Nanotechnology & Engineering with a thermal decomposition furnace following GB/T17664-1999. The dissolved biochar was obtained by washing 15g each biochar in 500 mL sddH_2_O and passed through a 0.65 μm filter. And the dissolved organic carbon of biochar was detected by total organic carbon analyzer (TOC-VCPH, Shimadzu, Japan).

We also examined the volatile organic compounds (VOCs) and water-extracted polycyclic aromatic hydrocarbons (PAHs) of ABC, BBC, MBC and PBC. The composition of VOCs was determined by the solid phase microextraction method (SPME, 75 μm Carboxen/PDMS, Supelco, Bellefonte, PA, USA) and then characterized by pyrolysis-gas chromatography/mass spectrometry (GC-MS, 7890-5975C, Agilent Inc., USA). Details of the parameters of VOC measurement were reported in Li, Chen [24]. The water-extracted PAH_16_ in biochar was detected according to Rogovska, Laird [25]. An Agilent 7890GC-7000C triple quadrupole MS (MDL: 1 μg/kg) was used to analyze the samples. Standard substances purchased from Sigma-Aldrich (USA) and Sangon Biotech (China) were used to generate external standard curves for quantifying samples.

### 2.2. Nematode culture and physiological and biochemical indexes

Wild-type N2 (Bristol) nematodes were obtained from NanKai University, and the worms were grown on nematode growth medium (NGM). The *Escherichia coli* OP50 strain was prepared as the food source for the nematodes. The strain was cultured in LB liquid medium for a day before it was used to feed the worms. We applied OP50 at a concentration of 10x to the NGM. All worms were synchronized by 1x lysis buffer and transferred to M9 buffer to grow on NGM in the presence of different biochars. The preparations of media and buffers were made according to the protocol which was originated from Brenner [26] and modified by He [27]. Ten milligrams of biochar was placed in the center of each petri dish (6.5 cm) with the help of an Oxford cup (d = 0.5 cm). A 1 mL buffer of age-synchronized eggs (~500) of the wild type *C*. *elegans* was placed on the biochar surface in each petri dish.

Body length was not measured until the *C*. *elegans* reached the day 1 adult stage (56 h). The media were washed off of the worms with 2 mL M9 buffer. Then, the nematodes were killed with gentle heat, and 1 mL of worms was drained randomly, and their body length was recorded [28]. Nematodes were observed under an Olympus SZX16 microscope, and nematode morphology and body lengths were measured by cellSens Dimension software.

Reproduction assays were carried out after morphological observations. One L4-stage worm was transferred to each petri dish (3.5 cm) containing NGM and 50 μL OP50 in LB, totaling 25 worms for each treatment. Nematodes grew at 25°C for 72 h, after which the number of worms at all stages except eggs was recorded.

L4 stage worms were also used in the SOD assay according to a previous reference [29]. We first collected the worm samples and ensured that there was 1 mL, 2 mL, and 4 mL of ice-cold PBS buffer in the tubes containing nematodes. Then, glass grinders were utilized to homogenize the nematodes and obtain the protein on ice (4°C). Then, the tubes were centrifuged at 4°C at 5,000 xg for 5 min, and any supernatants were discarded from each sample. The diluted samples were used with the SOD Assay Kit (Sigma) to determine the activity of superoxide dismutase in *C*. *elegans* by Multiskan Spectrum (Thermo Scientific, 450 nm).

According to Mishra [30], lifespan was evaluated by the rate of worms developed into the L4 larval stage. After 40 hours transfer from the synchronized eggs, the worms would be reaching the L3/L4 molt stage, and the number of L4 worms could be counted and recorded during the 44–55 h. Every hour afterward, the number of worms was recorded until they all reached the L4 stage.

In chemotaxis assays, 1 mL buffer of the wild type *C*. *elegans* are placed in the center of the 9 cm NGM petri dish with the peanutshell biochar on one side and each other biochars (MBA/ABC/BBC) on the other side (Madžarić, Kos [31], Baiocchi, Dillman [32]). Worms in regions with a radius of 1.5 cm around each Oxford cup were counted at 2 h, 4 h, 6 h, 12 h, 24 h, 36 h and 48 h. During the test, five replicates (Petri dishes) were conducted. The details and experimental setup of the biochar chemotaxis test protocol are shown in S1 Fig.

### 2.3. Transcriptome sequencing

The duration of biochar exposure prior to RNA-sequencing is about 56 hours. Once the age-synchronized worms grew to young adult worms, they were harvested and stored at -80°C after quick freezing by liquid nitrogen for the RNA-sequencing. Total RNA of young adult worms was extracted by TRIzol reagent (Sangong, Shanghai). Then, the RNA was purified by silicon membrane. RNase-free water was used to dissolved RNA. RNA sequencing was carried out by Sangong Biotech. Briefly, RNA was quantified and qualified by a NanoPhotometer^®^ spectrophotometer (IMPLEN, CA, USA) and an RNA Nano 6000 Assay Kit with the Agilent Bioanalyzer 2100 system (Agilent Technologies, CA, USA).

A total amount of 3 μg RNA per sample was used as input material for the RNA sample preparations. Sequencing libraries were generated using the NEBNext ^®^ Ultra^™^ RNA Library Prep Kit for Illumina^®^ (NEB, USA) following the manufacturer’s recommendations, and index codes were added to attribute sequences to each sample [28]. Then, 3 μl USER Enzyme (NEB, USA) was used for size selection and adaptor ligation of cDNA at 37°C for 15 min followed by 5 min at 95°C before PCR. PCR was performed with Phusion High-Fidelity DNA polymerase, universal PCR primers and Index (X) Primer. Finally, PCR products were purified (AMPure XP system), and library quality was assessed on the Agilent Bioanalyzer 2100 system. To analyze the sequences, HISAT2, RSeQC, Qualimap and BEDTools software was utilized. GO and KEGG enrichment analysis were performed to identify which DEGs were significantly enriched in GO terms and metabolic pathways at *p*-value≤0.05 compared with the whole-transcriptome background. GO functional enrichment and KEGG pathway analysis were carried out by Gene Ontology database (http://www.geneontology.org/), R (ClusterProfiler) and KAAS (https://www.genome.jp/tools/kaas/) [33].

### 2.4. Quantitative RT-PCR

According to the physiological and biochemical variation of *C*. *elegans* in this study, eight target genes (*col-184*, *col-140*, *col-129*, *lys-7*, *ilys-5*, *cdk-8*, *sod-1*, *perm-4*) were found from wormbase and previous studies [34–37]. Then they were validated by quantitative real-time PCR (qPCR). EasyScriptct^®^ One-Step gDNA Removal and cDNA Synthesis Super Mix were used to synthesize cDNA. A Thermal Cycler^®^ was used for quantitative PCR performed with 2×Trans Start^®^ Top/Tip Green qPCR Super Mix. Three independent biological replicates were applied for each gene, and the expression levels of each gene were normalized on the basis of the reference gene *pmp-3* [38]. The relative expression ratio of target genes was calculated using the ^ΔΔ^Ct method.

### 2.5. Statistical analysis

The statistics were analyzed by SPSS 22 using t-tests if variances were unequal. Moroever, we compared the differences among different groups through univariate analysis (ANOVA). If the data did not pass the variance homogeneity test, Games-Howell tests were applied in one-way ANOVA. The mean values and standard errors of the mean are presented below and were calculated using SPSS 22.

Chemotaxis indexes (CIs) were used to evaluate the results for the worms in biochar chemotaxis experiment and were calculated as follows:

Chemotaxis index (CI) = (worm count in PBC-treated group) − (worm count in MBC/PBC/ABC-treated group)/(worm count in PBC-treated groups) + (worm count in MBC/PBC/ABC-treated groups).

A CI value of 0.5 to 1.0 indicated that the majority of worms were in/around the PBC-treated group, while a CI of -1.0 to -0.5 indicated that most worms were in/around the other biochar (MBC/ABC/BBC-treated) groups. Additionally, a CI value near zero indicated that there was no difference between the number of worms in both treatments.

## 3. Results

### 3.1. Properties of different biochars

The lignocellulosic biomass of raw materials and physicochemical properties of biochars are described in Tables 1 and 2. The four raw materials differed in the percentages of hemicellulose, lignin and fiber, with the lowest NDF and ADF for maize straw and the highest for aspen sawdust. All biochars were typically alkaline, with pH values ranging from 8.76 to 9.92. Moreover, the value of DOC in PBC, namely 29.46%, was the highest, followed by ABC, BBC, and MBC of 7.66 mg/L, 5.50 mg/L, and 7.43 mg/L, respectively. The biochars also possessed a range of 11.45–19.36 m^2^/g specific surface area, 0.89–4.77 nm porosity, 3.0%-16.2% ash, 83.05%-96.34% fixed carbon, 5.50–29.46 mg/L dissolved organic carbon and 0.66%-1.43% volatiles.

**Table 1:** The lignocellulosic biomass of raw material for biochar.

Materials	NDF (%)	ADF (%)	Hemicellulose (%)	Lignin (%)	Fiber (%)
Aspen sawdust	87.15	73.28	13.87	29.34	61.86
Bamboo powder	78.74	56.06	22.68	18.50	44.74
Maize straw	55.38	32.09	23.29	6.90	25.03
Peanut shell	70.81	58.35	12.46	30.81	48.29

**Table 2:** Physicochemical properties of biochars made from different raw materials.

Biochar	pH	Specific surface area (SSA,m^2^/g)	Porosity (nm)	Ash Content (%)	Fixed carbon (%)	DOC (mg/L)	PAH_16_ (μg/kg)	Volatiles (%)
ABC	8.76	17.36	2.03	4.10	94.78	7.66	62.48	1.12
BBC	9.84	11.45	4.77	3.00	95.57	5.50	93.58	1.43
MBC	9.32	18.09	1.77	16.20	83.05	7.43	87.20	0.75
PBC	9.92	19.36	0.89	3.00	96.34	29.46	95.83	0.66

Most of the PAH contents of the biochars could be attributed to Pyr, which accounted for 49.0–59.7%. Finally, we observed that the VOC profiles from the four biochars varied greatly (Table 3). The most positively identified volatile compounds, seven, were in PBC compared to the three compounds identified in ABC and MBC, as well as only one compound identified in BBC (S2 Fig). Additionally, the results also suggested that the VOCs were almost the same among ABC, MBC and BBC, with similar silicon compounds and retention times.

**Table 3:** Profiles of products observed in ABC, MBC, BBC and PBC.

Materials	No.^a^	Volatile organic compounds	Molecular formula	Retention time (min)	CAS No.
PBC	1	2,2-Dimethyldecane	C_12_H_26_	12.339	17302-37-3
	2	2,2,4,6,6-pentamethyl-Heptane	C_12_H_26_	12.885	13475-82-6
	3	7H-Dibenzo[b,g]carbazole,7	C_21_H_15_N	13.603	3557-49-1
	4	3,7-Dimethyldecane	C_12_H_26_	15.822	17312-54-8
	5	2,3,7-Trimethyldecane	C_13_H_28_	16.099	62238-13-5
	6	4,7-Dimethylundecane	C_13_H_28_	18.236	17301-32-5
	7	3-Ethyl-3-methylheptane	C_10_H_22_	18.048	17302-01-1
ABC	8	Octamethylcyclotetrasiloxane (D4)	C_8_H_24_O_4_Si_4_	13.603	556-67-2
	9	Ethyl hexylketone	C_9_H_18_O	17.689	925-78-0
	10	Decamethylcyclopentasiloxane	C_10_H_30_O_5_Si_5_	21.009	541-02-6
BBC	11	Octamethylcyclotetrasiloxane (D4)	C_8_H_24_O_4_Si_4_	13.636	556-67-2
MBC	12	Octamethylcyclotetrasiloxane (D4)	C_8_H_24_O_4_Si_4_	13.660	556-67-2
	13	Ethyl hexyl ketone	C_9_H_18_O	17.641	925-78-0
	14	Decamethylcyclopentasiloxane	C_10_H_30_O_5_Si_5_	21.026	541-02-6

^a^ The numbers refer to the peaks in the spectra of S2 Fig.

### 3.2. Developmental observation of *C*. *elegans* under different biochars

**Fig 1:**
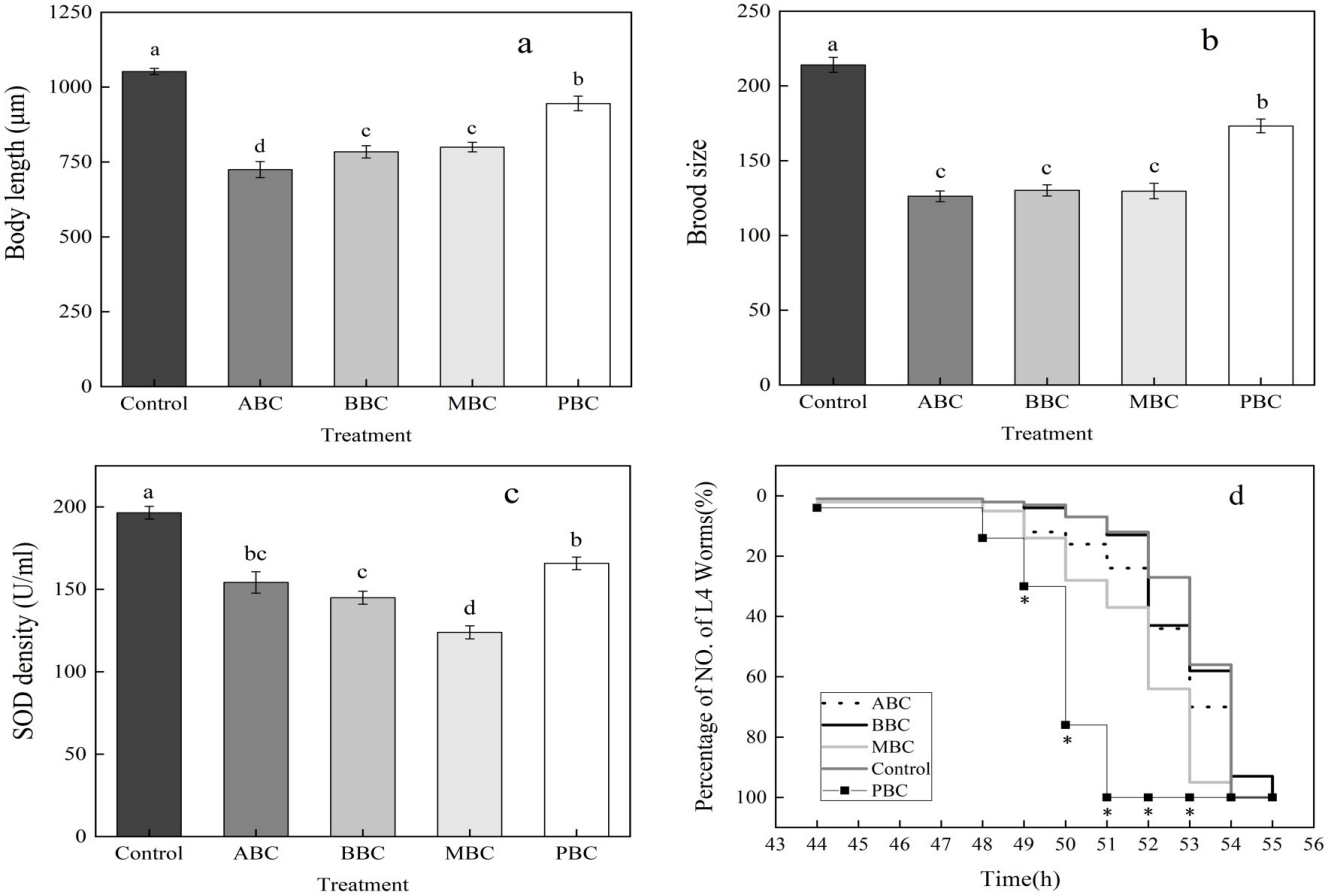
Brood size (a), body length (b), SOD activity (c) and lifespan (d) of *C*. *elegans* under the biochar treatments. Different lowercase letters indicated significant differences between treatments (Tukey’s test, *P* < 0.05); the stars indicated significant differences between peanut-treated group and each other biochar (ABC/BBC/MBC-treated) group.

The brood size, body length, SOD activity and the lifespan (L4 reaching time) were evaluated in the four biochar (ABC, BBC, MBC and PBC)-treated groups in comparison with the water control group. The results showed that exposure to these biochars significantly decreased the body length of *C*. *elegans*. The rates of decrease were 31.12%, 25.48%, 23.99% and 10.16% for ABC, BBC, MBC and PBC exposure, respectively (Fig 1a). Among the treatments, worms in ABC had the shortest average body length, with values of 724.6±26.7 μm, which was significantly shorter than that of worms in PBC (945.2±24.3 μm) and the control (1052.1±10.3 μm). Worms in the BBC and MBC treatments were similar in body length, with mean values of 799.7±15.8 μm and 784.0±20.4 μm, which were also significantly shorter than the control value.

The brood size refers to the number of hatched worms during the observation period among treatments. The brood size was significantly decreased following biochar exposure, and the reduction reached 39.44%, 39.21%, 41.07% and 19.10% in the MBC-, BBC-, ABC- and PBC- exposed groups, respectively, compared to the control group (Fig 1b).

The results also demonstrated that the SOD density in the biochar-treated group was significantly lower than that in the control group (Fig 1c). The SOD assay results showed that the control group had the highest SOD activity, which was 196.5±3.86 U/mL. The MBC treatment resulted in the lowest SOD activity (123.9±3.89 U/mL), which was nearly 1/3 of the control level. ABC, BBC and PBC decreased the SOD activity to 154.2±6.49, 145.0±3.95 and 165.81±3.82 U/mL, respectively.

According to Fig 1d, the *C*. *elegans* exposed to peanut shell biochar had a shorter lifespan than those exposed to other biochars or the control group. Almost all worms took 51 hours to developed into young adult in PBC treatment. However, most worms took 53–55 hours from synchronized eggs in the other biochars (MBC, BBC, ABC) treatments and the control to reach adulthood.

**Fig 2:**
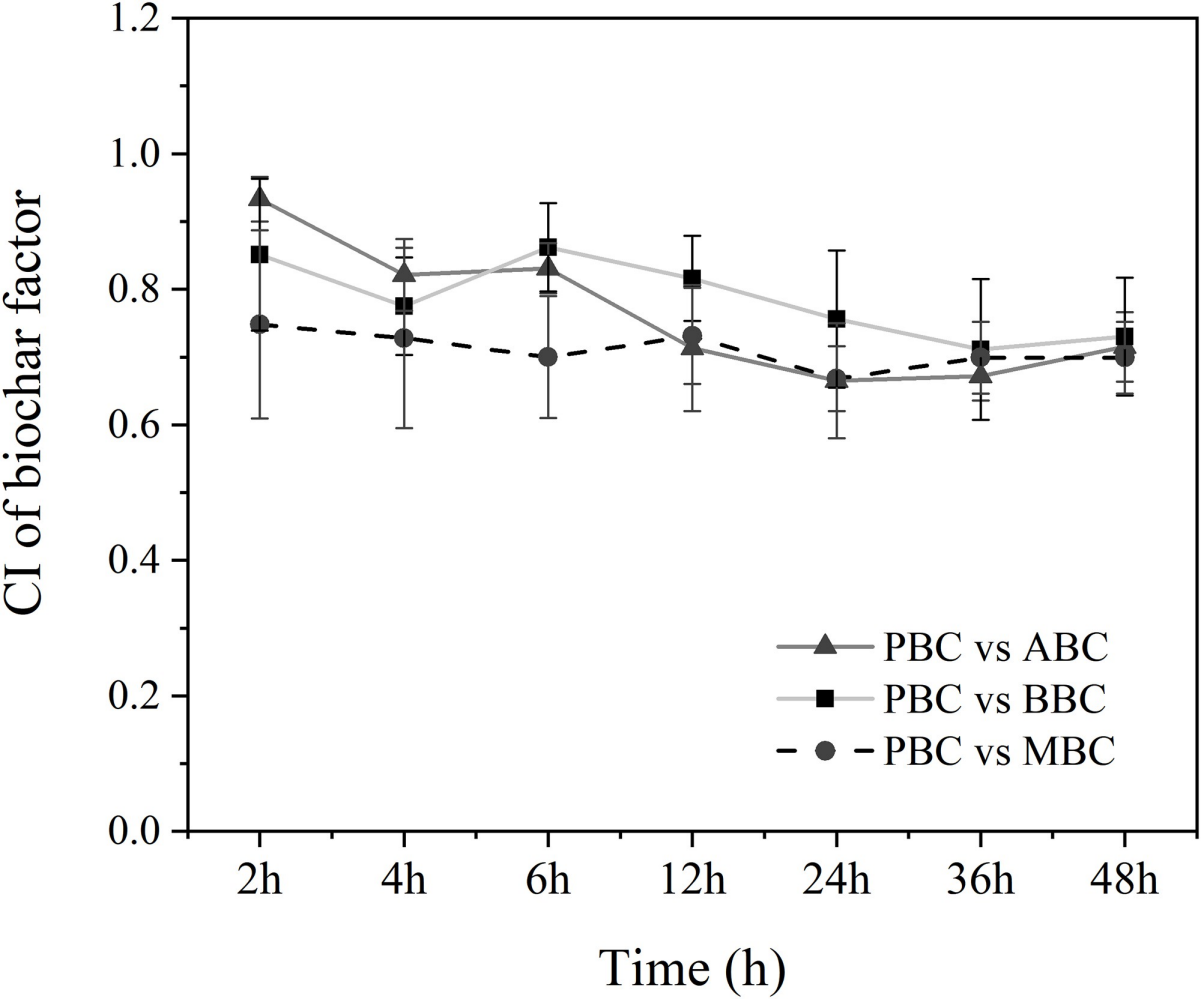
Chemotaxis index (CI) values of *C*. *elegans* among different biochar treatments.

Moreover, *C*. *elegans* shows chemotaxis to PBC compared to other biochars (Fig 2). The variation of CI between PBC and each other biochars were 0.66 ~ 0.93 during the cultivation period, which indicated that more nematodes moved around PBC than ABC/MBC/BBC after 48 h of treatment. However, the statistically significance of chemotaxis indices were not significant between PBC-treated and ABC/BBC/MBC-treated group.

### 3.3. RNA sequencing analysis of *C*. *elegans* in different biochars

**Fig 3:**
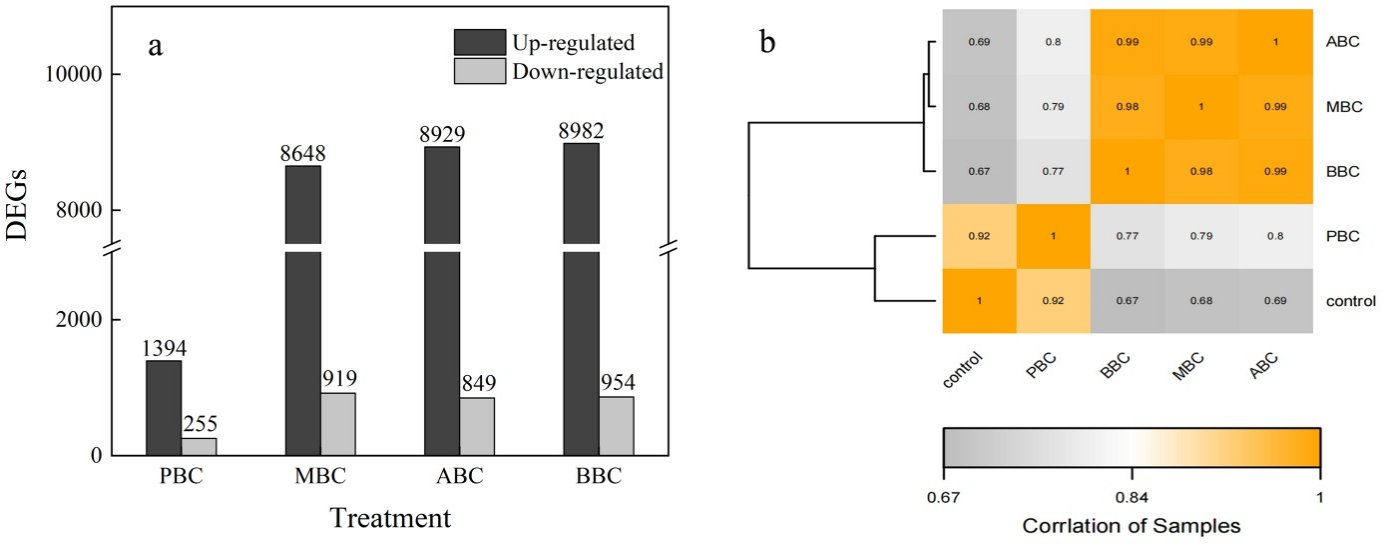
Differentially expressed genes of *C*. *elegans* under biochar treatments (a) and correlation heatmap between treatments (b). The number above each column indicated the differentially expressed genes of *C*. *elegans*.

To determine the mechanisms of the depressive response, transcriptome sequencing and analysis were conducted on *C*. *elegans* exposed to ABC, MBC, BBC and PBC for 56 h. To identified differential expression among various groups of *C*. *elegans*, false discovery rate (FDR-) adjusted *p*-values <0.05 and absolute fold change (>1.0) among treatments were regarded as thresholds. Compared to the control, 9778 (8929 up- and 849 downregulated genes), 9936 (8982 up- and 954 downregulated genes), 9567 (8648 up- and 919 downregulated genes) and 1649 genes (1394 up- and 255 downregulated genes) were differentially expressed following ABC, BBC, MBC and PBC treatments, respectively (Fig 3a).

**Fig 4:**
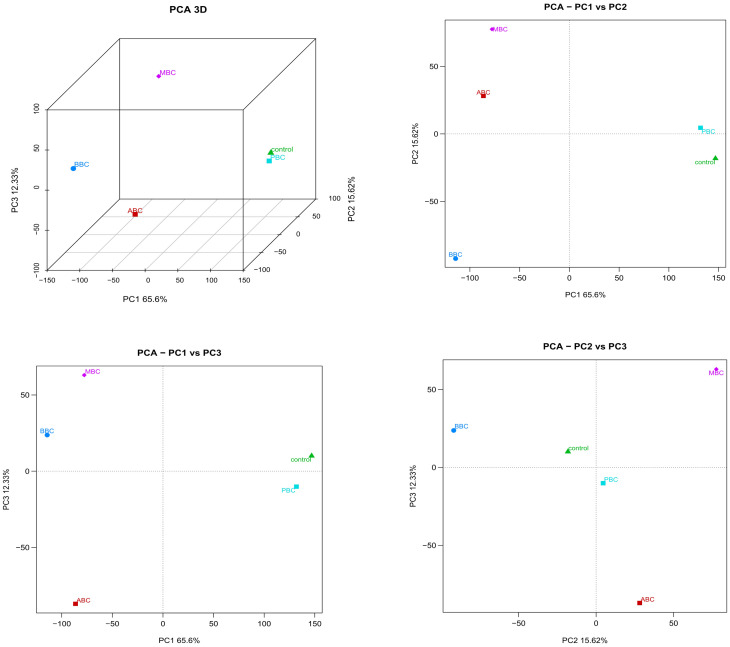
Principal component analysis of DEGs for *C*. *elegans* exposed to different biochar treatments.

A hierarchical clustering of correlation heat-map demonstrated the expressed gene data of ABC/MBC/BBC were grouped together, while the PBC/Control cluster were grouped together (Fig 3b). Moreover, according to correlation analyze and principal component analysis (PCA), it was confirmed that worms in the ABC, BBC and MBC treatments were very different from those in the PBC and control (Fig 4, S3 Fig). The first, second and third principal components explained 65.6%, 15.62% and 12.33% of the variation, respectively.

### 3.4. Functional analysis of DEGs in biochar-exposed *C*. *elegans*

**Fig 5:**
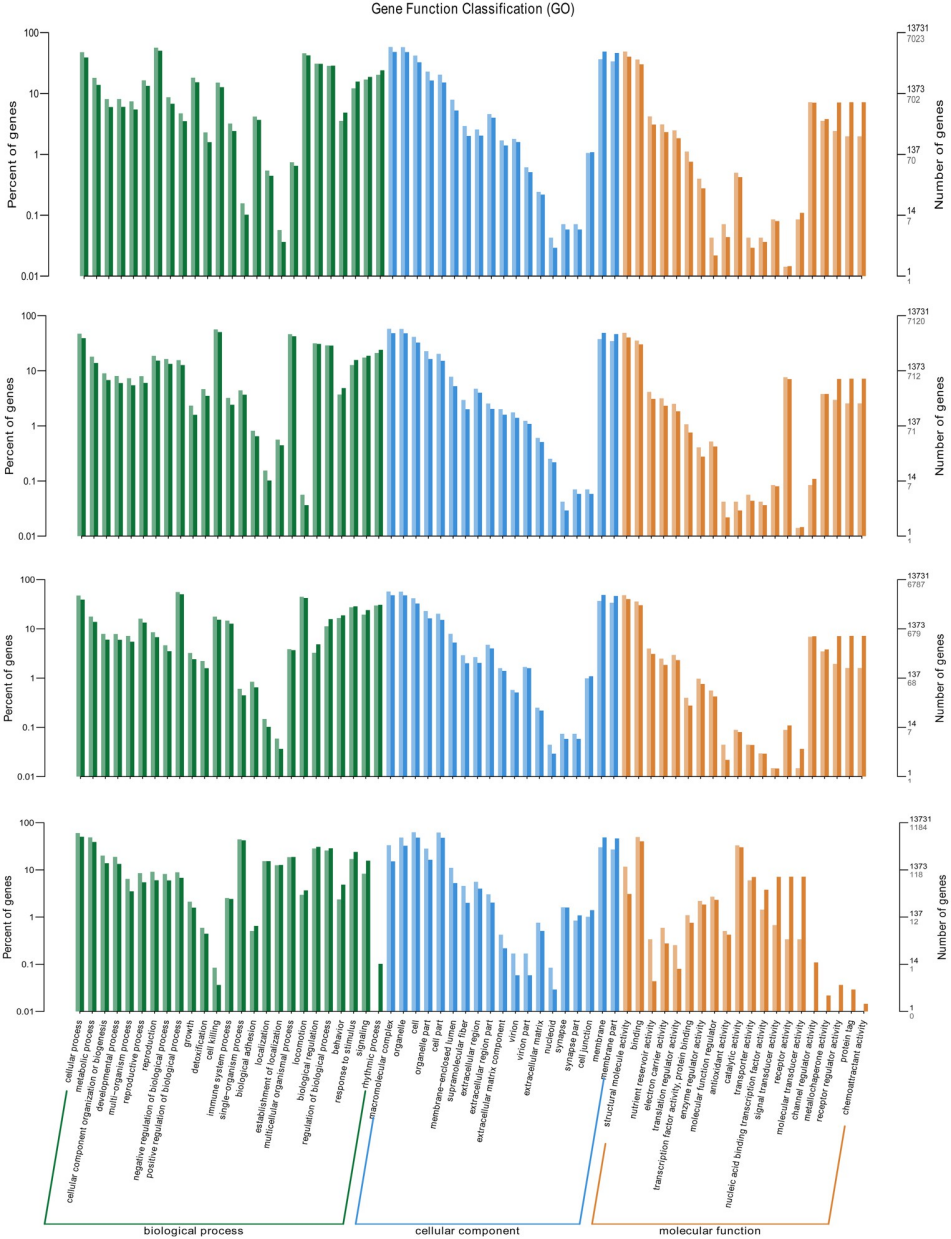
Major gene ontology (GO) terms for C. *elegans* exposed to different biochar treatments. Dark/light colour of columns and numbers in the figure and right axis represent the differentially expressed genes and total genes.

The identified DEGs under the biochar treatments were further annotated with GO analysis and classified into biological process (BP), cellular component (CC), and molecular function (MF) categories. Every DEGs were annotated into different sub-categories belonging to the following three GO categories in biochar treatments (Fig 5).

According to Fig 5, in BP sub-categories, the majority DEGs were related to organic substance metabolic process, metabolic process, cellular metabolic process and etc. Cellular metabolic process and translation were different among treatments. Among the CC sub-categories, the majority of GO terms were grouped into cell, intracellular, nuclear, endoplasmic reticulum, etc. Protein complex, ribonucleoprotein complex and pseudopodium were differentially expressed among biochars during environmental information processing. In MF sub-categories, genes for binding activity, catalytic activity and protein binding were in the top three terms, in which structural molecule activity and structural constituent of cuticle were more in ABC, BBC and MBC than the PBC groups.

Moreover, scatter plots displayed the GO enrichment among the different biochar groups (S4 Fig). The rich factors of ABC-, BBC- and MBC-treated groups were 0.5~0.8, which were higher than the value of PBC-treated group (0.1~0.5). And the GO enrichment analysis demonstrated that the cell and cellular progress was the largest cluster, followed by cell, cell part, intracellular and etc.

**Fig 6:**
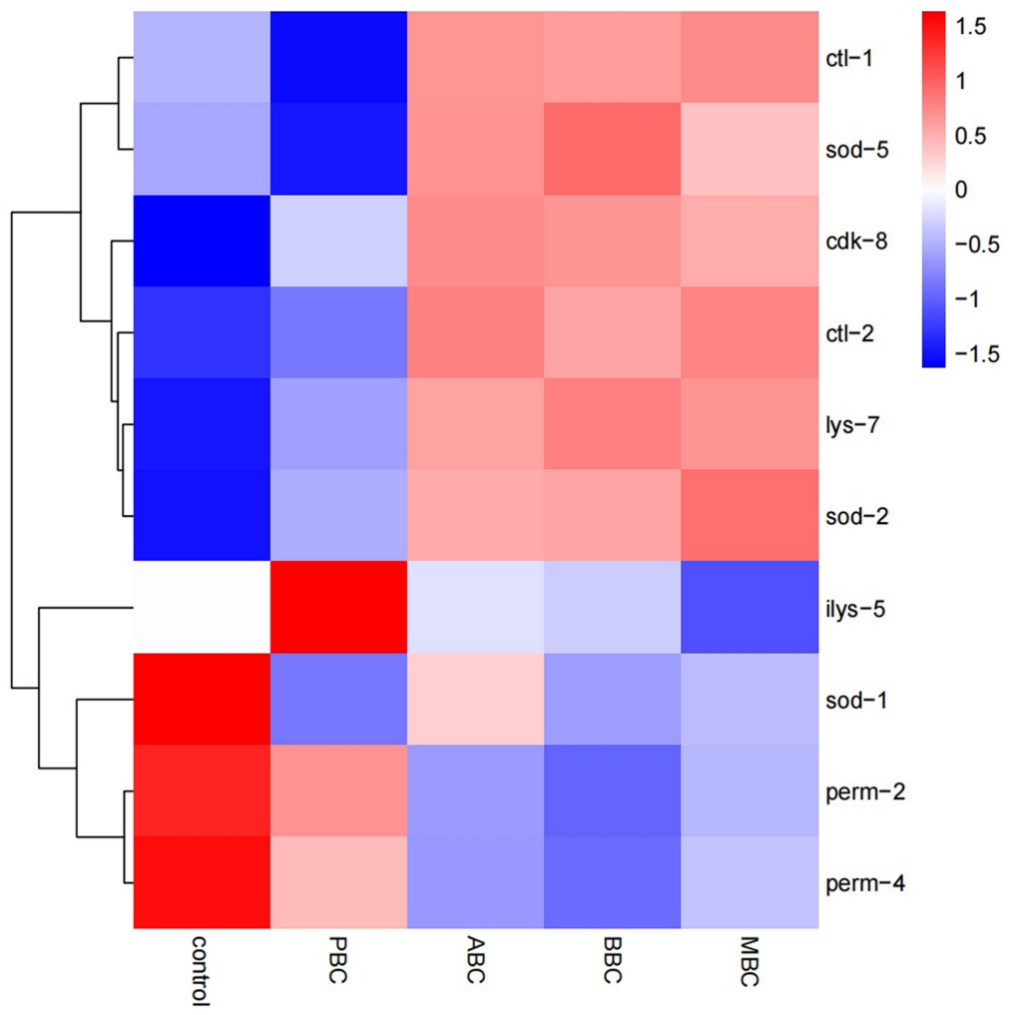
Clustering analysis heatmap for the top ten DEGs in *C*. *elegans* exposed to different biochar treatments. Different colors represent the Z values of the DEGs abundance after standardization.

Hierarchical clustering analyses were performed on the heat-map of 20 most-expressed genes in the four biochar treatment groups (Fig 6). Among the genes analyzed, there was a significant separation of the ABC-, BBC- and MBC-treated groups from the control and PBC-treated groups.

**Fig 7:**
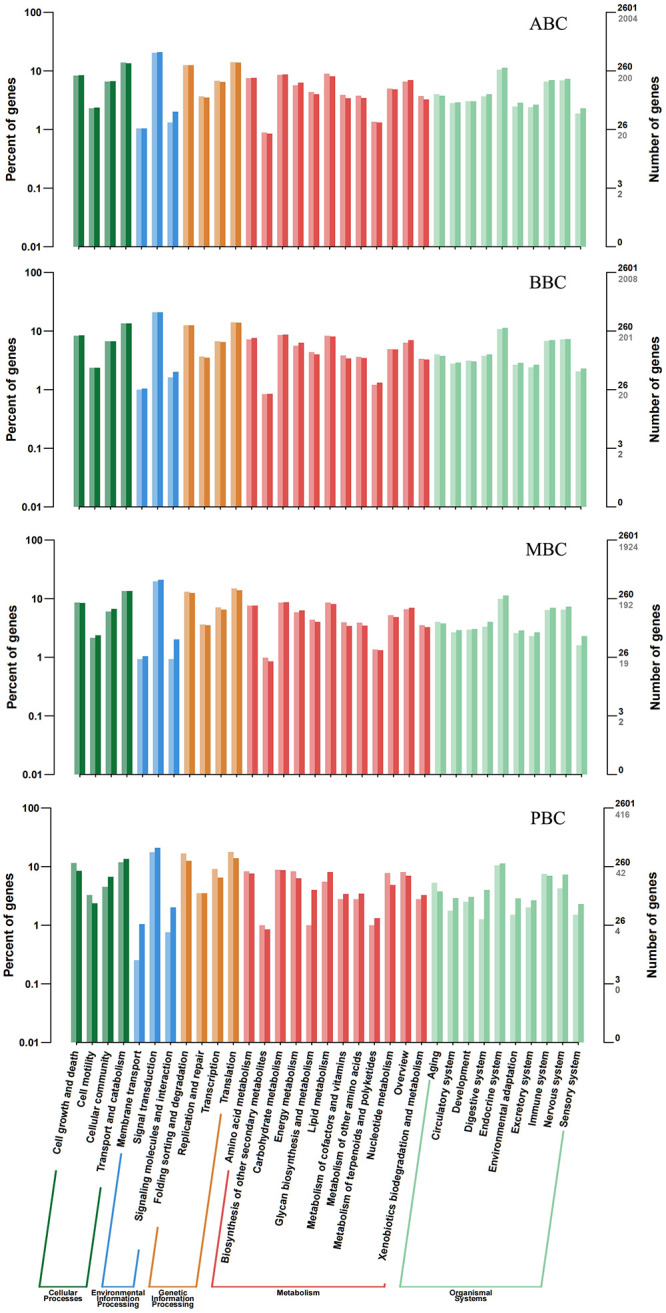
KEGG pathway classification for *C*. *elegans* exposed to different biochar treatments. Dark/light colour of columns and numbers in the figure and right axis represent the differentially expressed genes and total genes.

In addition, based on KEGG pathway mapping, the identified DEGs were classified into the following five KEGG functional categories (cellular processes, environmental information processing, genetic information processing, metabolism and organismal systems). A summary of the DEGs are demonstrated in Fig 7. According to further KEGG analysis, the top sub functional categories were N-Glycan biosynthesis, MAPK signaling pathway, TNF signaling pathway, ribosome biogenesis in eukaryotes and glycerophospholipid metabolism in ABC, BBC and MBC. Results showed that worms in the PBC treatment showed maximum alterations in proteasome, pyrimidine metabolism, RNA degradation, ribosome and apoptosis compared to the control. However, these progressed were less important in ABC, BBC and MBC groups, indicating the nematodes in other biochars varied in different ways.

### 3.5. Analysis of target genes related to the differential phenotypes of *C*. *elegans*

**Fig 8:**
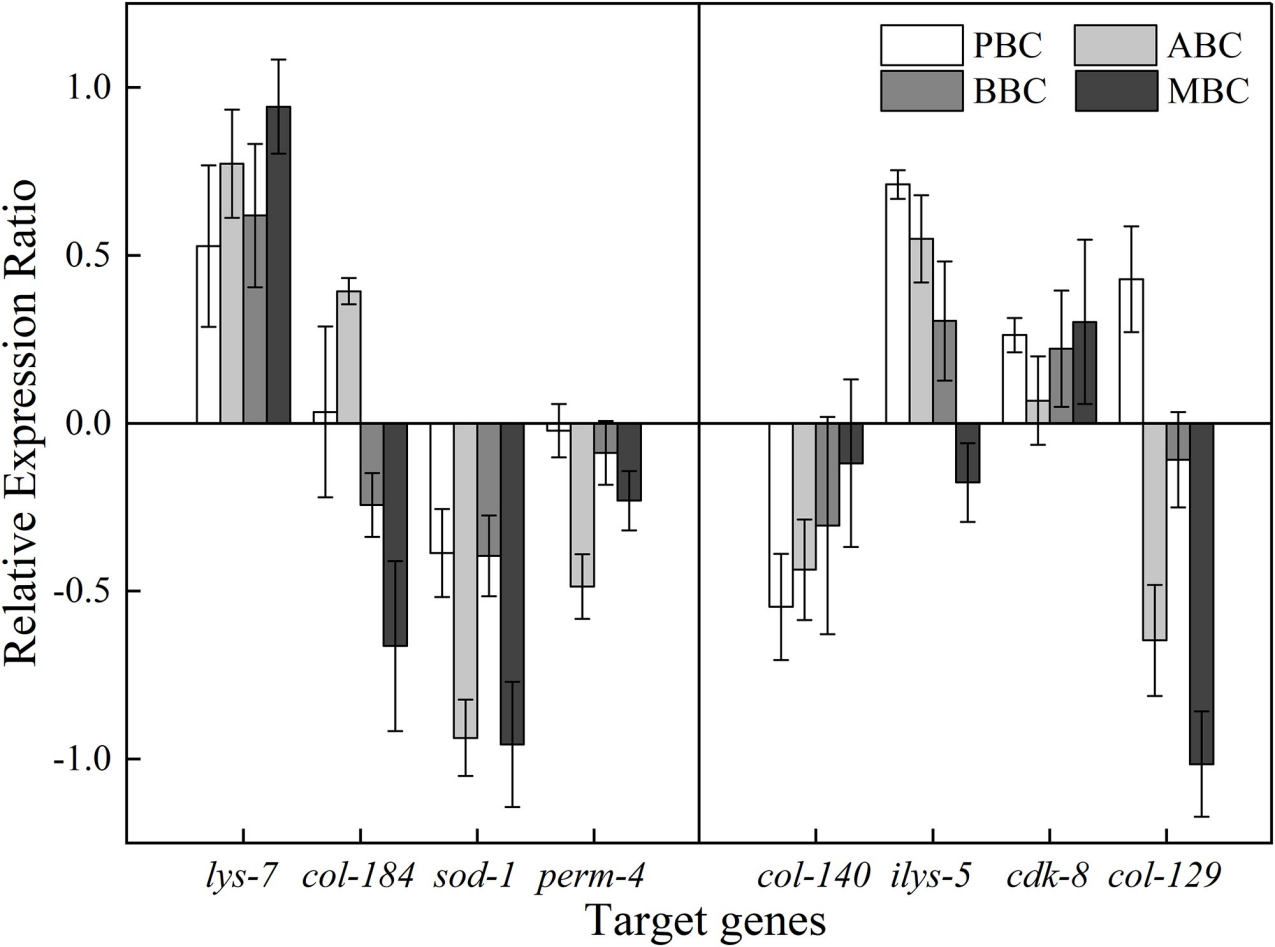
Different gene expression patterns of *C*. *elegans* exposed to different treatments using qPCR.

Five extracellular structure component-encoding genes (*col-184*, *col-140*, *col-129*, *lys-7*, *ilys-5*), one life span-related gene (*cdk-8*), one oxidative stress-related gene (*sod-1*) and one eggshell vitelline layer-related gene (*perm-4*) were confirmed through quantitative real-time PCR. The permeable eggshell-encoding gene *perm-4* was significantly downregulated under biochar treatment, whereas extracellular structure component-encoding genes such as the *col*-gene family (*col-129*, *col-140*, *col-184*) and *lys-7* were differentially regulated compared to those in the control group. Life span-related genes (*cdk*-8) were expressed slightly up-regulated under biochar treatment compared with nematodes in the control, while the expression of oxidative stress-related genes (*sod-1*) was down-regulated (Fig 8).

## 4. Discussion

### 4.1. Development of *C*. *elegans* exposed to different biochars

Biochars were obtained from a variety of agricultural and forest sources so that the physicochemical components, PAHs, and VOC profiles would be correspondingly different. This study showed that the four raw materials differed in lignocellulosic biomass, and the biochars varied in ash content, pH, porosity, etc. (Table 2), similar to the differences in physico-chemical properties reported in Ronsse, van Hecke [39] and Kan, Strezov [40].

The total PAH_16_ content of the four biochars was less than 0.1 mg/kg, which was much lower than the European (3 mg/kg, Communities S.O.O., 2011) [41] and American (6 mg/kg, Khair et al., 2000) standards [42]. The result was similar to those of De la Rosa, Sánchez-Martín [43] and Hale, Lehmann [44], who observed significantly lower PAHs in crop residue biochars developed under low oxygen and 450–600°C pyrolysis than in biochars derived from animal manure.

As previous studies mentioned that mobile organic compounds were produced during the pyrolysis process and identified widely in biochars [45, 46], our research confirmed that both the contents and composition of VOCs were significantly varied among biochars made from different biomasses. Moreover, worms in our study showed a preference for biochar derived from peanut shells over those derived from aspen, bamboo and maize straw (Fig 2). As Baiocchi, Dillman [32] and Busch, Kammann [47] noted that nematodes can detect specific mobile compounds by using their sensory cues, the fact that the highest number of VOCs was identified from PBC might play an important role in worm attraction.

Given the exposure of nematodes on the surface of biochars, our study demonstrated that biochar shortened the life span of *C*. *elegans* and decreased body length and brood size, which was different from the results obtained with biochar applied to farmland soils. Zhang, Li [7] found no significant difference in free-living nematode abundance among 2.4–48 t/ha biochar treatments after a 7-month application. Pressler, Foster [48] also demonstrated that one year of biochar amendment did not contribute to the negative effects of soil nematodes in a crop field. Thus, without soil buffer capacity, biochar-exposed treatment may result in environmental stress of the growth of *C*. *elegans*.

Moreover, there is an obvious difference between PBC-exposed nematodes and ABC/BBC/MBC-exposed worms. PBC-exposed C. *elegans* had milder decreases in body length, brood size and SOD activity relative to other biochar-exposed worms. Dissolved organic matter (DOM) may be an important factor to alleviate adverse impact on *C*. *elegans*. Hoss [49] implied that DOM could potentially be an additional carbon source for bacterial cells and serving as food for nematodes. Lieke [50] found fulvic acid played an essential role in the stability of environmental persistent free radicals (EPFRs) and may protect aquatic organism species from the harms of persistent free radicals. The dissolved organic carbon of PBC was 29.46 mg/L, which was approximately 4–5 times than that of other biochars in Table 2. Thus, higher DOM of PBC was assumed to alleviate the harmful environmental impact on *C*. *elegans*. Nonetheless, further investigation is needed to clarify the effect of DOM derived from biochar on nematodes.

### 4.2. Transcriptome analysis of C. *elegans* exposed to different biochars

Based on the phenotypic variation of C. *elegans* under biochar treatment, RNA-seq analysis can reveal the mechanism of transcription. According to gene ontology (GO) enrichment analysis, biochar mainly impacts genes involved in sensory processing, cuticle development, reproductive processes and immune system processes in C. *elegans* (Table 4). It was also found that several DEGs were obviously different among the four kinds of biochars (Table 5).

**Table 4:** Top common DEGs of *C*. *elegans* in response to different biochar exposure treatments.

DEGs	Regulation	Function	Location	References
*dpy-17*	Down	Predicted to be a structural constituent of the cuticle.	Expressed in the anterior ganglion; hyp7 syncytium; and lateral ganglion left neurons. Located in collagen and cuticulin-based cuticle extracellular matrix.	[51]
*sqt-3*	Down	Involved in cuticle development and collagen and cuticulin-based cuticle molting cycle.	Located in collagen and cuticulin-based cuticle extracellular matrix. Is expressed in hypodermis.	[52]
*rpl-18*	Down	Predicted to be a structural constituent of 40S and 60S.	—	[34]
*perm-2/4*	Down	Required for structural integrity of the vitelline layer of the eggshell.	Expressed in oocytes.	[35]
*sod-1/2*	Up	*sod-1* is involved in regulation of brood size, vulval development and response to oxidative stress; *sod-2* enables protein homodimeri- zation and superoxide dismutase activity to remove superoxide radicals.	*sod-1* is located in the cytosol and mitochondrion and expressed in several structures, including intestinal cells, somatic gonads, and the somatic nervous system; *sod-2* is located in the mitochondrial respirasome and expressed in the head and tail.	[53]
*cdk-8*	Up	Predicted to enable ATP binding activity, RNA polymerase II CTD heptapeptide repeat kinase activity and cyclin-dependent protein serine/threonine kinase activity.	Expressed in anchor cells and neurons.	[36]
*ctl-1/2*	Up	*ctl-1* enables catalase activity.	Located in peroxisomes.	[54]
		*ctl-2* enables catalase activity and is involved in determination of adult lifespan as well as peroxisome organization.		

**Table 5:** DEGs of *C*. *elegans* in response to different biochar exposures.

DEGs	Biochars	Regulation	Function	Main Description	References
*col-40/81/129*	PBC	Down/Up	Predicted to be a structural constituent of the cuticle	Expressed in the hypodermis	[34, 55]
		/Up			
*col-20/124/106*	MBC	Down	Predicted to be a structural constituent of the cuticle	Expressed in the hypodermis	[34, 55]
			Predicted to be a structural constituent of the cuticle.	Involved in cuticle develop- ment, collagen and cuticulin- based cuticle molting cycle.	
*col-20/124/140*	ABC	Down			[34, 55]
*col-119/122/140*	BBC	Down			[34, 55]
*bli-6*	PBC	Up			[56]
*ilys-5*	PBC	Up	Predicted to have lysozyme activity.	—	[57]
*icl-1*	PBC	Down	Predicted to have isocitrate lyase activity and malate synthase activity and be involved in determination of adult lifespan.	Localizes to the mitochondrion. ICL-1 appears to act down- stream of DAF-16 to influence lifespan.	[53]
*vit-3/4/5*	PBC	Up	Predicted to have lipid transporter activity and nutrient reservoir activity.	Localizes to cytoplasmic vesicles, vesicle lumen, and yolk granules. Expressed in embryonic cells, gonads, intestines, oocytes and the pseudocoelom.	[58, 59]
*vit-2*	MBC, ABC	Down	Predicted to have lipid transporter activity and nutrient reservoir activity.	Localizes to cytoplasmic vesicles,vesicle lumen, and yolk granules. Expressed in embry- onic cells, gonads, intestines, oocytes, and the pseudocoelom.	[58, 59]
*vit-2/6*	BBC	Down			[58, 59]
			Involved in determination of adult lifespan, embryo development.		
*sip-1*	ABC, BBC, MBC	Down			[53]
*irg-7*	ABC, BBC, MBC	Up	Predicted to have carbohydr- ate binding activity.	—	[60]
*daf-16*	ABC, BBC, MBC	Up	Involved in defense respon- ses to other organisms, regulation of dauer larval development, and regulation of transcription.	Localizes to both the cytoplasm and the nucleus, with the ratio between the two an important regulator of function.	[61, 62]
*dod-19*	ABC, BBC, MBC	Up	Involved in the innate immune response.	Localizes to the membrane raft.	[61, 62]
*lys-7*	ABC, BBC, MBC	Up	Involved in the defense response to Gram-negative and Gram-positive bacteria and the innate immune response.	Localizes to the apical part of cells and cytoplasmic vesicles. Expressed in intestines and neurons.	[57]
*sod-5*	ABC, BBC	up	Predicted to enable metal ion binding activity and super- oxide dismutase activity.	Expressed in amphid neurons.	[53]
*odr-4/10*	ABC, BBC, MBC	up	Involved in olfactory behavior, positive regulation of chemotaxis and response to odorant.	Located in chemosensory neurons and somatic nervous system.	[63]

Although *C*. *elegans* seemed not to sense and/or be attracted to the ABC/BBC/MBC, the *odr-4* and *odr-10* genes upregulated significantly in ABC/BBC/MBC treatment. These genes were important to encode a tail-anchored transmembrane protein of ODR-10, which mediates *C*. *elegans* chemotaxis to volatile odors [63]. It seemed that the worms in ABC/BBC/MBC treatment attempt to strengthen their olfactory receptor, even if there is very few volatile odors detected from ABC/BBC/MBC. The regulation of odor sensory processing of nematode is complex, therefore, further confirmation of the adaptive process is needed.

Cuticle collagen synthesis is a complex process throughout life, and it has been shown that a multigene family of approximately 154 members could encode collagen-like proteins [34]. The most important genes were involved in the modification of the worm exoskeleton belong to the *col*, *vit* and *rpl* gene families [64]. Specific target genes varied among biochars. For instance, *col-40/81/129* was associated with PBC amendment, while *col-20/124/106*, *col-20/124/140* and *col-119/122/140* were related to the MBC, ABC and BBC treatments, respectively.

Biochar significantly impacts the reproductive process, resulting in a smaller brood size. *Perm-2* and *perm-4* were reported as key genes for eggshell formation [35, 64] and were the top downregulated genes under all biochar treatments in this study (Table 4). Recent studies stated that *perm-2* and *perm-4* encode the vitelline layer to protect the embryonic soluble factors from leaking to extracellular matrix [58]. The process is as follows: proteins PERM-2 and PERM-4 hinge to each other and then stabilize on CBD-1 to form the vitelline layer, and inhibiting any of them makes the adhesive component of the eggshell surface absent, which might prevent the connection alternatively between uterine secretions and the outer eggshell layer. Therefore, losing this protective barrier would cause not only soluble factor leakage but also embryonic death [65]. As a result, the down-regulating *perm-2/4* genes could be associated with the brood size inhibition in biochar treatment.

Other genes, such as members of the *sqt*, *bli*, and *dpy* gene families encoding major protein components of the cuticle [51, 52, 56]. Furthermore, Frankie found that the mutants of these target genes are significantly shorter and fatter than the wild type [56]. Thereby, variations of *sqt-3*, *bli-6* and *dpy-17* gene expression would be associated with shorter body length in biochar-treated groups [66, 67].

Additionally, the downregulation of *sip-1* and upregulation of *irg-7* were also detected in the ABC, BBC and MBC treatments (Table 5) and are associated with embryo development [61, 62] and carbohydrate binding activity [60, 68], indicating more inhibition of brood size in the other three biochars than in PBC.

Genes involved in rhythmic processes were also detected via transcriptome analysis. The *cdk-8* gene was upregulated in all biochar treatments (Table 4). According to Steimel, Suh [36], CDK-8 is required in neurons for correct navigation of commissures and interneuron axons in the ventral cord. Further research demonstrated that the *cdk-8* mutant had commissure navigation defects [61, 69], suggesting that *C*. *elegans* had to upregulate *cdk-8* to repress a specific pathway to ensure proper commissure navigation [36]. Moreover, the *daf-16*, *sip-1* and *icl-1 genes* are also associated with determinate worm lifespan by encoding DAF-16, which is a necessary component of promotor orthologs (FOXO1, FOXO3, and FOXO4) [53, 70]. In this study, the downregulation of the *sip-1* gene was found in the ABC, BBC and MBC treatments (Table 5), while the downregulation of *icl*-1 was found in the PBC treatment only, indicating that different biochars regulated different genes to shorten the nematode lifespan.

The *C*. *elegans* genome encodes catalase (CTL) and superoxide dismutase (SOD) to eliminate mitochondrial and cytoplasmic ROS. According to previous reports, *sod-1* encodes cytosolic Cu/Zn SOD, *sod-2/5* encodes mitochondrial (Mn-SOD) isoforms [37, 53], and *ctl-1/2* encodes an unusual cytosolic isoform or a typical peroxisomal catalase [37]. Corroborated by Schaar, Dues [71], this study demonstrated that the SOD activity, brood size and lifespan of C. *elegans* were significantly changed in the ABC, BBC and MBC treatments in comparison with those in PBC and the control (Table 5), which confirmed that the interaction among *ctl-1* and *sod-2* may cause a lower brood size and an abnormal lifespan under oxidative stress.

In summary, *C*. *elegans* was significantly underdeveloped in body growth, reproduction and life span under different biochar treatments, and these changes were associated with key gene variation under biochar exposure. Genes in the *col*, *dpy*, *and bli* families were differentially expressed, affecting coding of the major proteins of the cuticle and disrupting the development of the exoskeleton; genes in the *perm*, *sip* and *irg* families were associated with eggshell formation during embryonic development. Other genes, such as members of the *daf*, *sip*, *icl*, *lys*, *ctl* and *sod* families, were involved in life-span regulation and immune systems. Our study explored the effects of different biochars on *C*. *elegans* and the related mechanisms, providing insights for the use of biochar in agricultural management.

## 5. Conclusion

Biochar made from different agricultural wastes varied in physicochemical properties, total PAH_16_ contents and VOCs. We tested the phenotypic variation, selective behavior and transcriptome mechanisms of C. *elegans* in response to four different biochars. This study noted that most worms were located near the biochar made from peanut shell after 56 h of exposure, indicating that PBC could produce more VOCs to attract C. *elegans* than ABC, BBC and MBC. Furthermore, RNA-seq analysis demonstrated that ABC, BBC MBC and PBC downregulated 849, 862, 919 and 255 C. *elegans* genes and upregulated 8929, 8236, 8648 and 1394 genes, respectively, in comparison to the control. The main target genes included those related to collagen (*bli-6*, *sqt-3*, *dpy-17* and *col* gene families), reproductive processes *(perm-2/4*, *sip-1* and *irg-7*), rhythmic processes (*daf-16*, *sip-1*, *icl*-1 and *cdk*-8) and immune systems (*sod-1/2/5*, *ctl-1/2* and *lys-7*). Variations in those genes might have contributed to a significantly shorter body length and life span as well as a smaller brood size. Overall, our study clarified the response of *C*. *elegans* to different biochars and revealed the underlying mechanisms of biochar interactions with nematodes.

## Supporting information

S1 Fig Experimental setup of the chemotaxis assay. PBC was placed in A, and each other biochar (MBC/ABC/BBC) was places in B, respectively. C point refers the center of the petri dish. Gray color indicated the scoring region for counting *C*. *elegans*.
(TIF)

S2 Fig Chromatograms of biochars derived from peanut shell, aspen, bamboo and maize straw, corresponding to PBC, ABC, BBC and MBC, respectively. The numbers refer to the volatile organic compounds in Table 3.
(TIF)

S3 Fig Correlation of *C. elegans* expressed genes between biochar treatments and the control. (TIF)

S4 Fig Major gene ontology (GO) terms for *C*. *elegans* exposed to different biochar, respectively. Rich factor was the ratio of significant to annotated DEGs.
(TIF)

S1 Table Lists of differentially expressed genes of *C*. *elegans* for each biochar. (XLSX)

S2 Table All identified GO categories of *C*. *elegans* for each biochar. (XLSX)

S3 Table All identified KEGG categories of *C*. *elegans* for each biochar. (XLSX)
