# Supplementary figures and images for: The effects of different biochars on Caenorhabditis elegans and the underlying transcriptomic mechanisms
Source: PLoS One. 2023 Sep 22;18(9):e0284348. doi: 10.1371/journal.pone.0284348 (PMC10516431; doi:10.1371/journal.pone.0284348)

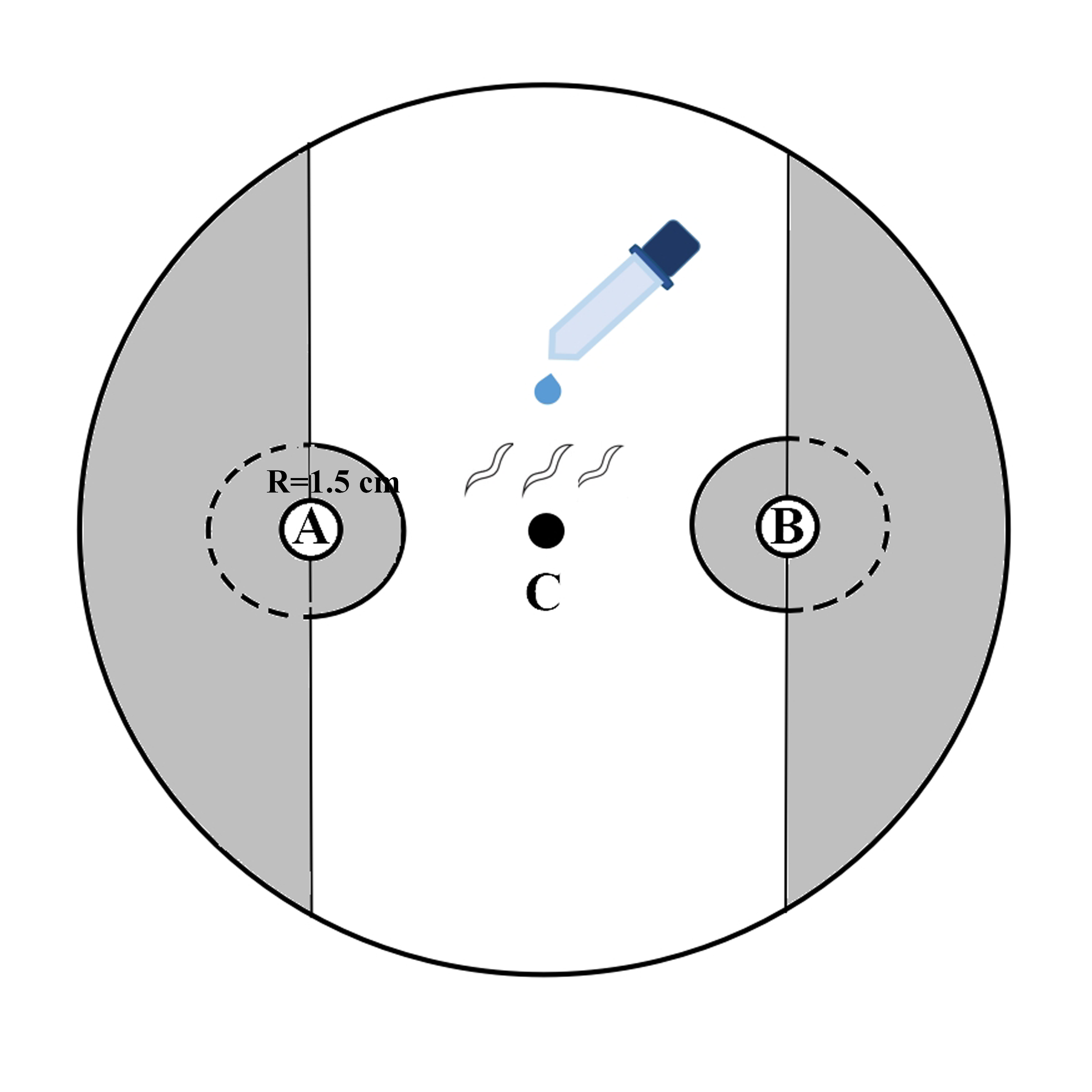

Supplement: S1 Fig — PBC was placed in A, and each other biochar (MBC/ABC/BBC) was places in B, respectively. C point refers the center of the petri dish. Gray color indicated the scoring region for counting C. elegans. (TIF) [file pone.0284348.s001.tif]

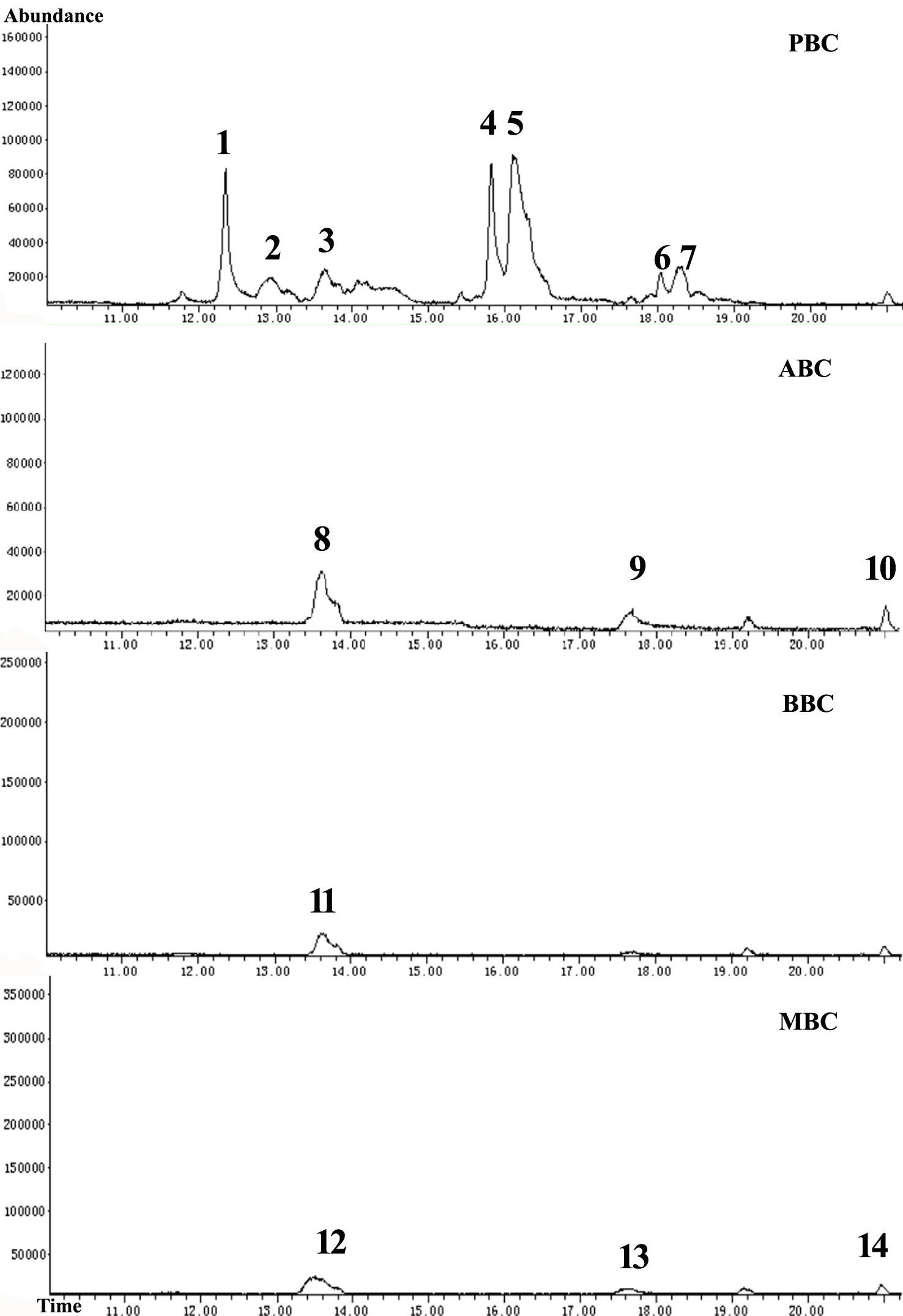

Supplement: S2 Fig — The numbers refer to the volatile organic compounds in Table 3. (TIF) [file pone.0284348.s002.tif]

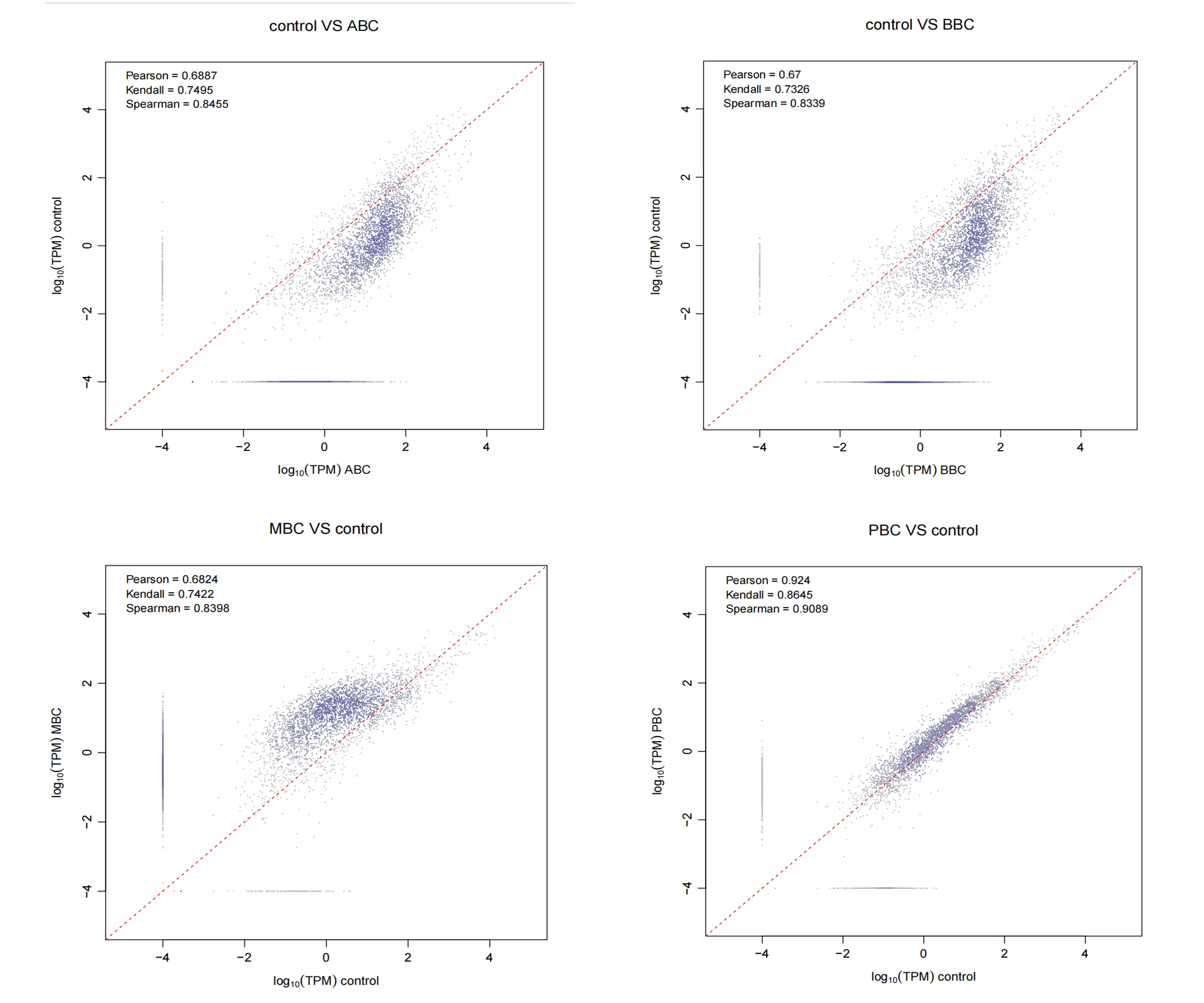

Supplement: S3 Fig — (TIF) [file pone.0284348.s003.tif]

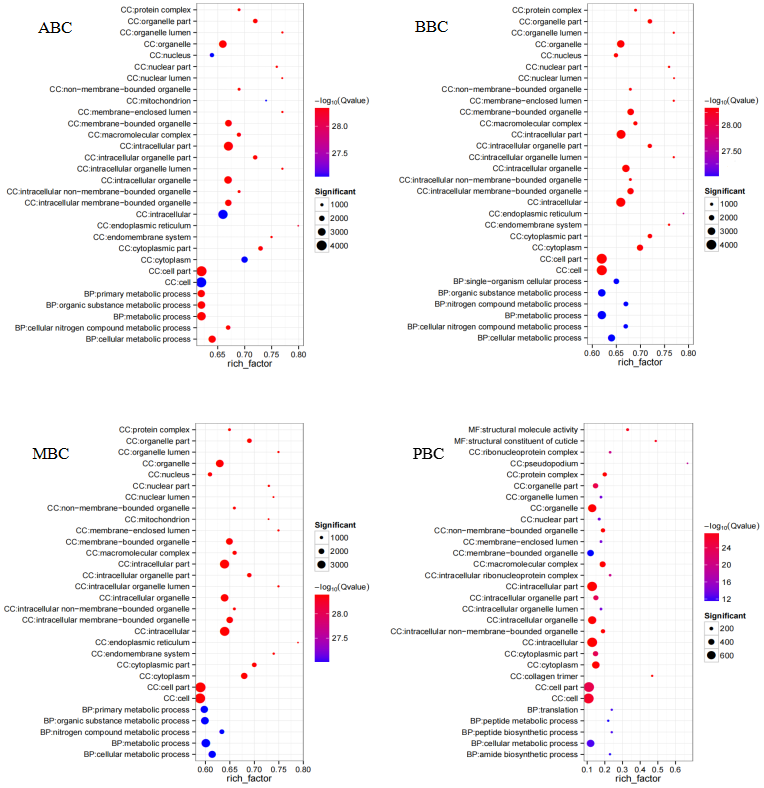

Supplement: S4 Fig — Rich factor was the ratio of significant to annotated DEGs. (TIF) [file pone.0284348.s004.tif]
